# Interdecadal shift in the spring Southern Annular Mode intensifies its lagged influence on Antarctic summer sea ice

**DOI:** 10.1093/nsr/nwag314

**Published:** 2026-05-27

**Authors:** Juan Dou, Xiangzhou Song, Renhe Zhang

**Affiliations:** Key Laboratory of Marine Hazards Forecasting, Ministry of Natural Resources, Hohai University, Nanjing 210098, China; College of Oceanography, Hohai University, Nanjing 210098, China; Key Laboratory of Marine Hazards Forecasting, Ministry of Natural Resources, Hohai University, Nanjing 210098, China; College of Oceanography, Hohai University, Nanjing 210098, China; Department of Atmospheric and Oceanic Sciences and Institute of Atmospheric Sciences, Fudan University, Shanghai 200438, China

**Keywords:** Southern Annular Mode, Antarctic sea ice, interdecadal shift, Pacific–South American pattern, Amundsen Sea low

## Abstract

Antarctic sea ice has recently shifted to a new low-extent regime, yet the underlying mechanisms remain uncertain. This study reveals an interdecadal shift in the spring Southern Annular Mode (SAM) pattern around 1998, after which its lagged influence on summer Antarctic sea ice intensified, particularly in the Weddell and Ross Seas. After 1998, the SAM exhibits stronger wave-like structures, featuring an intensified Pacific–South American (PSA)-like pattern that drives regional sea-ice loss. In the Weddell Sea, the positive SAM‑related anticyclonic circulation induces upper-ocean warming that promotes summer ice melt. However, in the Ross Sea, a deepened Amundsen Sea low enhances offshore ice export and positive ice‑albedo feedback, increasing summer ice loss. This interdecadal SAM shift is likely modulated by tropical variability, as the increased co-occurrence of a positive SAM with La Niña strengthens PSA-mediated processes. These findings highlight the nonstationary nature of tropical–polar linkages in recent Antarctic sea-ice lows.

## Teaser

Since the late 1990s, the Southern Annular Mode, a major atmospheric circulation pattern around Antarctica, has exerted a stronger delayed influence on summer sea ice. Its more frequent interaction with La Niña events has led to greater ice loss in the Weddell and Ross Seas via atmospheric teleconnections.

## INTRODUCTION

Antarctic sea ice plays a vital role in shaping the global climate system by modulating surface albedo, ocean–atmosphere heat exchange, and deep-water formation. Following a slight long-term expansion until 2014, the Antarctic sea-ice extent (SIE) declined abruptly in 2016 [1], with record-low minima recurring in February (the annual sea-ice minimum month) in subsequent years [2–6]. This retreat is exceptional over the past century and may indicate a regime shift toward a persistent low sea-ice state [7–9]. These extreme anomalies increase the urgency of elucidating the drivers behind Antarctic sea-ice variability. Unlike the Arctic, where sea-ice decrease is driven largely by anthropogenic warming, Antarctic sea-ice variability results from complex atmosphere-ocean interactions [10–12]. These interactions have contributed to highly irregular evolution of Antarctic sea ice observed in recent decades [13–17].

Among the atmospheric drivers, the Southern Annular Mode (SAM) is the predominant mode of climate variability in the extratropical Southern Hemisphere and strongly influences Antarctic sea ice [18,19]. The SAM is typically described as a zonally symmetric annular pattern characterized by opposing pressure anomalies between mid-latitudes and Antarctica [20–22]. Through this annular structure, the SAM regulates the strength of the circumpolar westerlies and exerts a timescale-dependent effect on sea ice. On interannual timescales, a positive SAM phase strengthens westerlies, drives northward Ekman transport, and can promote dynamic sea-ice expansion [23]. On longer multiannual to decadal timescales, persistently strengthened winds can induce Ekman pumping and the upwelling of warm Circumpolar Deep Water, which in turn melts sea ice [24]. Thus, the SAM’s net impact on sea ice depends on the interaction timescale between the atmosphere and ocean.

However, the SAM is not purely zonally symmetric in reality. Its observed patterns are often superimposed onto and interact with asymmetric circulation features [25], including tropically forced Rossby wave trains associated with the Pacific–South American (PSA) pattern [26] and quasi-stationary zonal wave-3 structures [27]. The interplay between the annular mode and these wave-like disturbances produces substantial zonal asymmetry and regionally heterogeneous sea-ice responses [28]. This zonal asymmetry in the SAM is manifested largely through variability in the Amundsen Sea low (ASL), which is a climatological low-pressure system in the South Pacific sector [29]. Changes in the depth and position of the ASL govern meridional wind anomalies and generate dipole-like sea-ice patterns between the Ross and Bellingshausen-Weddell Seas [30]. The spatial structure of the SAM also varies substantially across timescales, with seasonal anomalies exhibiting stronger zonal asymmetry than that in monthly or annual means and showing pronounced decadal fluctuations [28]. Recent decades have witnessed a shift in the SAM toward greater zonal asymmetry in certain seasons [31,32], which may fundamentally alter how the SAM interacts with Antarctic sea ice [19].

The influence of the SAM on Antarctic sea ice also exhibits pronounced seasonality. Its springtime influence is particularly important, as it can precondition the following summer sea ice through coupled ice–ocean feedbacks [33,34]. At the hemispheric scale, SAM anomalies during the sea-ice maximum period (August–November) can persist for up to 11 months [34]. Regionally, Guo *et al.* [35] reported that the increased interannual variability of Weddell Sea summer sea ice since the late 1990s is closely linked to the preceding spring SAM variability. The record-low summer SIEs in 2022 and 2023 have also been related to anomalous SAM conditions and associated ASL variability in the preceding seasons [14,16,36].

Despite these advances, an important gap remains in our understanding of the SAM–summer Antarctic sea-ice linkage. Previous studies have mainly emphasized either the regional Weddell Sea response to preceding spring SAM variability or the role of tropical Pacific forcing and PSA teleconnections [33,35], but it remains unclear whether the austral spring SAM itself underwent a structural transition in the late 1990s, and how this change may have altered its lagged influence on Antarctic summer sea ice on a broader circum-Antarctic scale. This study therefore addresses the following three related questions: (i) Did the spatial pattern of the spring SAM undergo a systematic transition around the late 1990s? (ii) If so, how does this change influence summer sea ice on a pan‑Antarctic scale? (iii) Is this pattern change modulated by tropical forcing, such as El Niño-Southern Oscillation (ENSO)?

To address these gaps, this study systematically investigates the interdecadal evolution of spring SAM spatial patterns and their effects on Antarctic summer sea ice. First, we apply k-means clustering to spring sea-level pressure (SLP) fields to classify SAM patterns and identify transitions. Second, we quantify the influence of each pattern type on pan‑Antarctic summer sea ice. Third, we diagnose the atmospheric and oceanic processes through which different SAM patterns drive regional ice anomalies. Finally, we examine the potential role of tropical forcing, particularly ENSO, in modulating the observed pattern evolution. By integrating pattern classification, statistical teleconnection analysis, and dynamical diagnosis, this work provides a mechanistic, pattern-based framework for understanding how evolving spring SAM variability shapes Antarctic summer sea ice under a changing climate.

## RESULTS

### Interdecadal shift in the spatial pattern of the spring SAM

**Figure 1.**
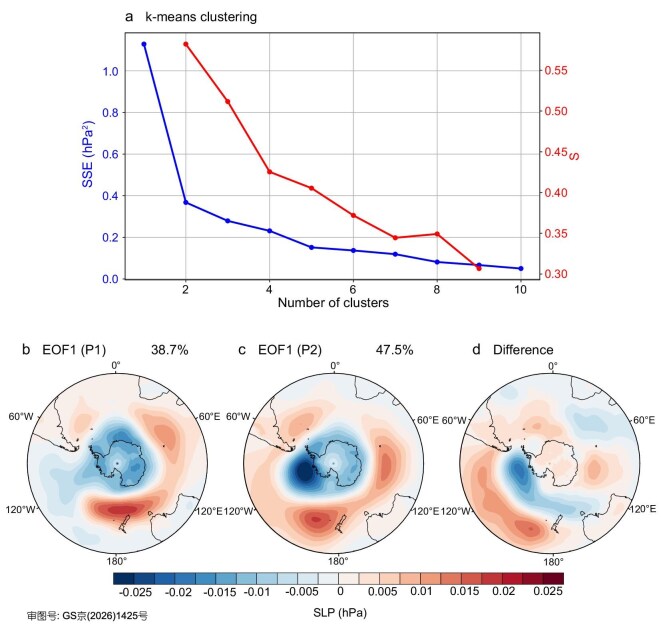
(a) Silhouette score (S, red line) and sum of squared error (SSE, blue line) from the k-means clustering analysis applied to the 21-year Rn-EOF-derived SAM patterns. (b–d) Spatial patterns of the leading EOF mode of SON SLP south of 20°S for (b) P1 (1979–1997), (c) P2 (1998–2023) and (d) the difference between the two periods (P2 minus P1). The percentage of explained variance is given in each panel title.

To determine the temporal evolution of the spatial structure of the austral spring (September–October–November, SON) SAM, we first apply the running empirical orthogonal function (Rn-EOF) method with a 21‑year sliding window. The resulting patterns and associated indices are consolidated into the positive SAM phase for subsequent clustering and analysis. The Rn-EOF analysis result reveals a gradual evolution of the SAM spatial pattern toward a more wave‑like configuration with an intensified ASL (Fig. S1). We then perform k‑means clustering on the spatial patterns derived from the Rn-EOF analysis. A two‑cluster solution is identified as optimal based on the maximum silhouette coefficient (S) and the inflection point in the sum of squared errors (SSE) (Fig. 1a). The clustering results divide the study period into two epochs: Period 1 (P1, 1979–1997) and Period 2 (P2, 1998–2023) (blue numbers in Fig. S1). The robustness of the detected transition point is confirmed by repeating the analysis with alternative sliding‑window lengths (17 and 19 years), which yield nearly identical results (figure not shown). To characterize the SAM patterns during each period, we perform separate EOF analyses for P1 and P2. The leading spatial patterns differ substantially between the two epochs (Fig. 1b–d). The leading EOF mode during the post‑1998 period explains a larger fraction of the total variance and exhibits a more notable wave‑like structure, characterized by an eastward‑shifted high‑pressure center near Australia, a deepened low over the Amundsen Sea, and an intensified high‑pressure anomaly over the South Atlantic. Together, these features indicate a more active PSA‑like teleconnection pattern and an intensified ASL. This spatial evolution is consistent with the Rn‑EOF results and indicates that since the late 1990s the SAM has become more strongly coupled with PSA‑like teleconnection signals. To further verify the PSA-like nature of this pattern, we calculate the correlation between the spring SAM index and the PSA index defined by Yuan and Li [18]. The correlation increases from 0.54 before 1998 to 0.90 after 1998, thus quantitatively confirming that the SAM pattern is more PSA-like in the later period.

### Strengthened lagged influence of the spring SAM on Antarctic summer sea ice

**Figure 2.**
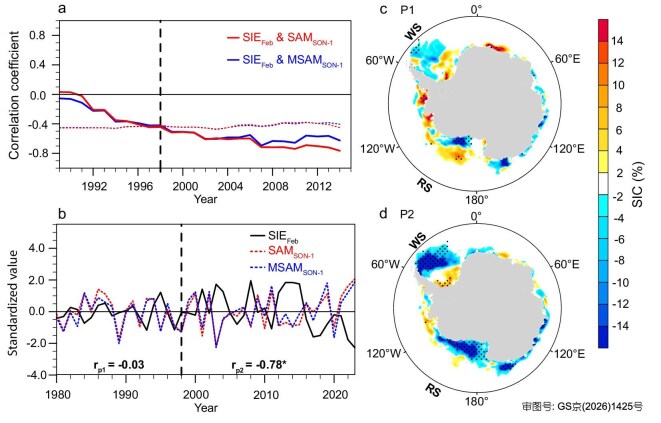
(a) Twenty-one-year sliding correlation between the February Antarctic SIE and the preceding spring (SON) SAM index (red line). Horizontal dashed lines indicate the 95% confidence level based on Student’s t test applied with effective degrees of freedom to account for autocorrelation. The vertical dashed line indicates the year 1998. (b) Normalized time series of the SON SAM index (red) and February Antarctic SIE (black) during 1979–2023. The blue lines in (a) and (b) denote the Marshall [20] SAM index. The correlation coefficients for the pre-1998 and post-1998 periods are r_p1_ = –0.03 and r_p2_ = –0.78*, where the asterisk (*) indicates statistical significance at the 99% confidence level. (c and d) Regression patterns of Antarctic SIC (%) in February onto the standardized SON SAM index for (c) P1 (1979–1997) and (d) P2 (1998–2023). Stippling indicates that the anomalies are statistically significant at the 95% confidence level. WS: Weddell Sea (0°–60°W), RS: Ross Sea (160°E–130°W).

The interdecadal change in the spatial structure of the SAM may fundamentally alter its influence on Antarctic sea ice. To identify the optimal response month, we examine lagged correlations between monthly SIE and the preceding spring SAM index. The strongest negative correlation occurs in February (Fig. S2), which also corresponds to the climatological minimum of Antarctic SIE. These factors motivate our focus on February. We then quantify the temporal evolution using a 21-year sliding correlation between the spring SAM index and the subsequent February Antarctic SIE (red lines in Fig. 2a and b). This sliding correlation begins to shift in the mid-1990s and strengthens markedly around 1998. Before 1998, the correlation remains weak, whereas after 1998 it becomes strongly negative (–0.78), significant at the 99% confidence level, indicating a substantially strengthened linkage. This result is robust to detrending and is reproduced using an alternative SAM index (blue lines in Fig. 2a and b) defined by Marshall [20]. Spatially, the SAM–Antarctic sea-ice concentration (SIC) relationship strengthens most prominently in the Weddell and Ross Seas (Fig. 2c and d). Their correlation coefficients increase sharply after 1998, from approximately –0.03 to –0.67 in the Weddell Sea and from –0.10 to –0.61 in the Ross Sea. The Weddell and Ross Seas together explain about 74% of the interannual variance in total Antarctic February SIE over 1998–2023. The record-low Antarctic SIE observed in February 2022 and 2023, following exceptionally strong spring SAM events (Fig. 2b), further supports the strengthened SAM–SIE relationship. To elucidate the underlying mechanisms, we next investigate how the spring SAM influences summer sea ice, with a particular focus on the Weddell and Ross Seas.

**Figure 3.**
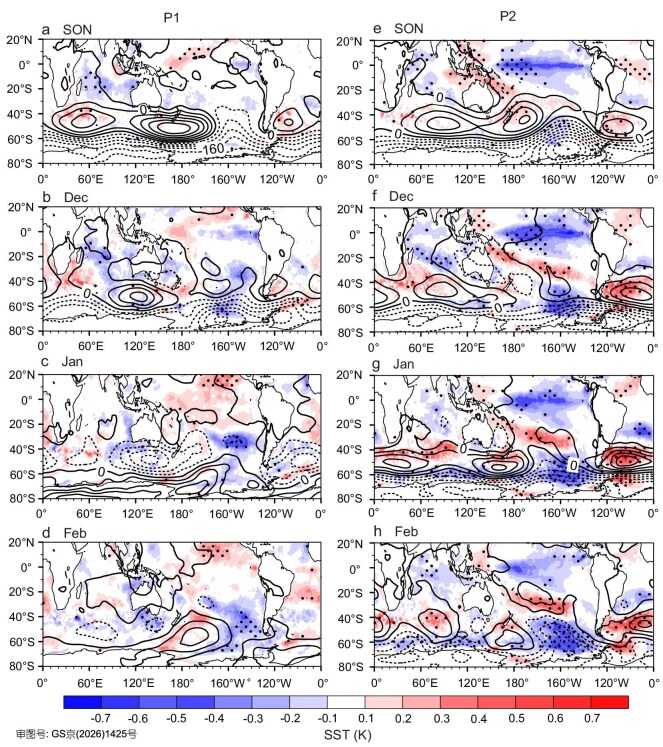
Regressions maps of the SST anomaly (shading; K) and 850-hPa geopotential height (contours; gpm) from SON to the following February onto the standardized SON SAM index during P1 (1979–1997; a–d) and P2 (1998–2023; e–h). The black dots indicate SST anomalies that are significant at the 95% confidence level.

The lagged influence of the spring SAM can be mediated through several interacting pathways within the ocean-sea ice system. Although the SAM, as an atmospheric mode, may decay rapidly, its effects can persist through slowly evolving boundary conditions. These include sea surface temperature (SST) anomalies, changes in sea-ice thickness, and shifts in upper-ocean heat content and stratification [12,19,37–39]. The evolution of atmospheric circulation and SST anomalies provides key insights into these pathways. Therefore, we first examine the conditions concurrent with the spring SAM and their evolution into subsequent seasons across the two epochs. As shown in Fig. 3, the SAM-related atmospheric and oceanic anomalies are weak prior to 1998, particularly over the South Atlantic–South Pacific sector. During this period, a relatively weak ASL is accompanied by modest cold SST anomalies confined to the South Pacific and weak warm anomalies in the South Atlantic. In contrast, the post-1998 period exhibits a notably amplified wave-like signature, characterized by a deepened ASL and two anomalous high-pressure centers east of New Zealand and over the South Atlantic, reflecting a stronger PSA-like teleconnection. Under this circulation regime, a pronounced tripole SST anomaly pattern emerges across the South Pacific and South Atlantic, persisting into the following summer. Although the SST anomalies near New Zealand at lower latitudes may have limited direct influence on sea ice, the dipole structure spanning the South Pacific to Atlantic sectors, which occurs closer to Antarctica, is likely more relevant for sea-ice variability. We therefore focus on this dipole pattern in the following analysis.

**Figure 4.**
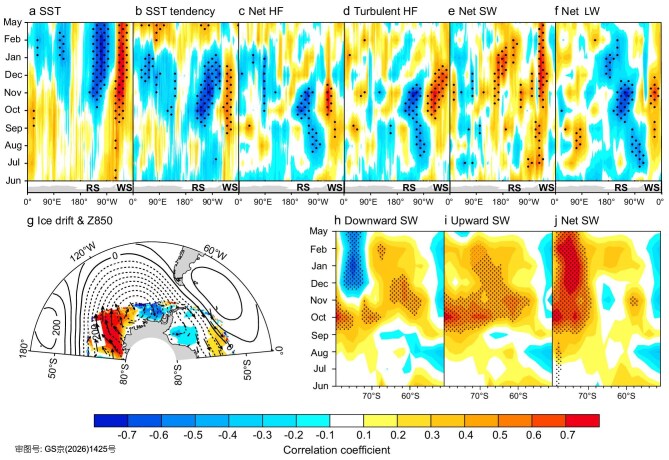
Physical mechanisms linking the spring SAM to summer sea ice during P2 (1998–2023), derived from correlations with the standardized SON SAM index. (a–f) Zonal mean (75°–45°S) time–longitude sections from June to the following May of (a) SST anomalies, (b) SST tendency (month-to-month change), (c) net surface heat flux (net HF), (d) turbulent heat flux (turbulent HF), (e) surface net shortwave radiation (net SW), and (f) surface net longwave radiation (net LW). (g) Correlations of the 850-hPa geopotential height (Z850, contours) and sea-ice drift (vectors) with its meridional component (shadings). (h–j) Meridional mean over the Ross Sea (RS, 180°–120°W) time–longitude sections of (h) surface downward SW, (i) surface upward SW, and (j) surface net SW. Positive flux anomalies indicate downward energy transport. Stippling denotes areas where correlations are significant at the 95% confidence level.

To elucidate how SAM-associated atmospheric circulation drives these SST changes after 1998, we investigate the time–longitude cross-sections of SST and surface flux anomalies averaged between 70°S and 50°S (Fig. 4a–f), correlated with the normalized SON SAM index during P2. The spatial correspondence between the SST tendency (Fig. 4b) and net heat flux anomalies (Fig. 4c) indicates that surface heat fluxes dominate the generation of SAM-related dipole SST anomalies (Fig. 4a) over the South Atlantic–South Pacific region. These surface flux anomalies are driven by the enhanced PSA-like teleconnection and the intensified ASL, which together modify the surface energy balance through several pathways. The anticyclonic circulation over the Weddell Sea suppresses cloud cover and increases the net surface shortwave radiation (SW) (Fig. 4e). On the northern flank of this anticyclone, easterly anomalies weaken the prevailing westerlies, reduce the turbulent heat loss (Fig. 4d) from the ocean and further promote SST warming. Additionally, on the eastern flank of the ASL, anomalous northerly winds transport warm, moist air into the Weddell Sea, thus increasing the downward longwave radiation (LW) (Fig. 4f) and favoring both warm SST anomalies and sea-ice reduction. Conversely, over the Ross Sea, the western flank of the intensified ASL promotes southeasterly wind anomalies that enhance cold, dry advection, thereby reducing downward LW. Concurrently, strengthened westerly winds over this region increase surface turbulent heat loss, collectively sustaining the cold SST anomalies. The persistent warm SST anomalies in the South Atlantic sector create favorable conditions for sea-ice reduction in the Weddell Sea. In contrast, before 1998, SAM-related air–sea coupling is weak, inducing only modest cold SST anomalies in the Ross Sea with a limited influence on sea ice (see Figs S2a and S3f).

The above results indicate that after 1998, the SAM, through its strengthened PSA-like signals, triggers a South Atlantic–South Pacific dipole SST pattern that persists into summer. The warm SST anomalies in the South Atlantic provide favorable conditions for the continued sea-ice reduction in the Weddell Sea. However, an apparent contradiction emerges in the Ross Sea. Notably, despite the presence of cold SST anomalies along the periphery of the ice edge, the observed SAM-related sea-ice anomalies decrease in the Ross Sea during the subsequent summer season (Fig. 2d). Cold SSTs typically favor ice growth, yet a reduction in sea ice occurs instead. This inconsistency raises the question of what processes drive summer sea-ice loss in the Ross Sea, despite the local cooling effect of SST anomalies after 1998.

From a dynamic perspective, the enhanced PSA-like configuration deepens the ASL, intensifying the meridional wind components over the Ross and Weddell Seas. These anomalous winds play a pivotal role in shaping sea-ice transport and exert a significant lagged influence on ice loss in the Ross Sea through wind-driven ice transport and ice–albedo feedback [14,17]. As shown in Fig. 4g, after 1998, intensified southerly winds along the western flank of the ASL drive stronger offshore ice advection, pushing sea ice away from the Ross Sea coast in spring. This process expands near-coastal open water, forming coastal polynyas. However, as air temperatures rise above freezing point from spring into summer, the ‘polynya ice factory’ becomes inefficient [14,17]. Near-coastal open water (south of 70°S), characterized by low albedo, absorbs more solar radiation and reflects less upward SW (Fig. 4h). Together with increased downward SW (Fig. 4i), this process enhances net downward SW at the surface (Fig. 4j), warming the upper ocean and strengthening the positive feedback that accelerates ice melt. Thus, in the Ross Sea sector, the combined effect of wind-driven ice export and ice–albedo feedback overwhelms the local SST cooling after 1998, resulting in a net reduction in sea ice in summer. In contrast, prior to 1998 (see Fig. S3g–j), the relatively weak ASL associated with the SAM generates only modest offshore wind-driven ice advection, which is insufficient to push sea ice far northward. During that period, the ice–albedo feedback is limited to near-coastal areas south of 75°S, leading to localized sea-ice loss that is largely offset by sea-ice increase north of 75°S, resulting in no significant net change across the overall Ross Sea (Fig. 2c).

To further validate these interpretations, we conduct a quantitative sea-ice budget analysis using Eq. (1). The SONDJF (September to February) sea-ice budget terms regressed onto the spring SAM index during 1998–2023 are shown in Fig. S4, including the total sea-ice concentration tendency, the dynamic component (advection and divergence), and the thermodynamic component related to melting/freezing. The budget decomposition provides quantitative support for the distinct regional mechanisms described above. Given that sea-ice drift is less reliable near the ice edge, the budget decomposition is most robust within the interior ice pack [40]. In the Weddell Sea, the reduction in sea ice is dominated by the thermodynamic component, confirming that oceanic and atmospheric heat fluxes play the leading role in driving summer ice loss. In the coastal Ross Sea, by contrast, the reduction in the sea-ice tendency arises from the combined effects of dynamic and thermodynamic processes. The dynamic component indicates that offshore ice transport is an important driver of the initial ice loss, while the thermodynamic component suggests subsequent amplification through enhanced upper-ocean heat uptake and ice melt. This finding is consistent with our previous analysis: dynamically driven offshore ice export in spring initiates coastal Ross Sea ice retreat, and the resulting open water enhances thermodynamic melt through ice–albedo feedback.

**Figure 5.**
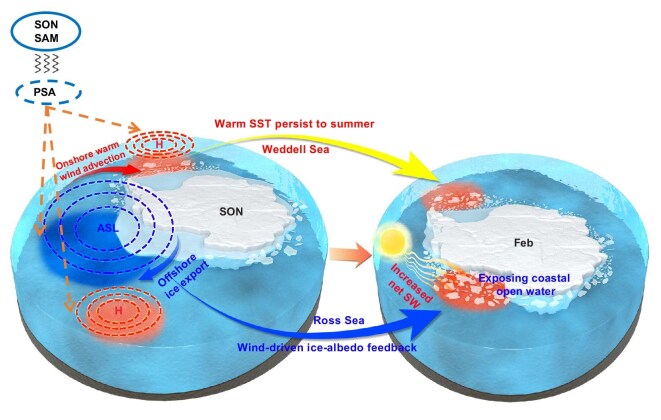
Schematic illustration of the lagged influence of spring (SON) positive SAM on February Antarctic sea ice over the Weddell Sea and Ross Sea during P2. These processes are primarily mediated by the Pacific–South American (PSA) pattern and the Amundsen Sea low (ASL). Solid (dashed) contours indicate anticyclonic (cyclonic) circulation systems, with ‘H’ denoting high-pressure centers. The underlying shadings represent SST anomalies (blue for cold, red for warm anomalies).

These results indicate that the intensified PSA-like teleconnection related to the positive SAM after 1998 drives summer sea-ice loss through two distinct pathways. In the Weddell Sea, strengthened air–sea interactions induce warm SSTs that persist through the summer, thereby promoting ice melt. Concurrently, in the Ross Sea, the deepened ASL enhances offshore ice export and triggers positive ice–albedo feedback, which accelerates upper-ocean warming and further ice melt (Fig. 5). Although the local processes differ, both regional responses originate from the same strengthened PSA–ASL circulation, with their contrasting expressions shaped by regional geography and climatology. In contrast, before 1998, the weaker SAM–PSA coupling and a less developed ASL result in limited preconditioning of summer sea ice in both regions.

### ENSO modulation of the spring SAM pattern shift

The interdecadal shift in the spring SAM pattern is likely influenced by tropical Pacific forcing. ENSO-related tropical convection can trigger southeastward-propagating Rossby wave trains that project onto the PSA pattern, and subsequently influence Southern Hemisphere circulation [14,41]. We first examine the linear relationship between ENSO and SAM during austral spring. The correlation between the spring SAM index and the Niño 4 index increases significantly from 0.12 during P1 to −0.40 during P2, indicating a stronger negative relationship in the later period. This is consistent with the findings of Yu *et al.* [42], who reported a strengthening of ENSO–SAM teleconnections after the early 1990s. This contrast is also evident in Fig. 3. During P1, positive spring SAM anomalies are not accompanied by clear ENSO or PSA signals, and the associated SST anomalies remain weak. In contrast, during P2, positive spring SAM anomalies coincide with La Niña-related forcing, a pronounced PSA-like component, and a more persistent SST dipole, with Weddell Sea warming and Ross Sea cooling extending into summer. This contrast suggests that, without ENSO-related forcing, the SAM alone may be insufficient to sustain a PSA-like asymmetry and the underlying SST anomalies.

However, the linear correlation alone is insufficient to capture the full complexity of the ENSO–SAM relationship, as out‑of‑phase SAM–ENSO combinations (positive SAM/La Niña or negative SAM/El Niño) can produce substantially stronger atmospheric impacts than either phenomenon alone [43,44]. Using a ±0.5 standard deviation (σ) threshold on the spring SAM and Niño 4 indices, we identify co-occurring SAM–ENSO anomalies at the event level. Positive austral spring SAM/La Niña events occurred in 1983, 1998, 1999, 2008, 2010, 2020, 2021, and 2022, while negative SAM/El Niño events occurred in 1982, 1991, 1994, 1997, 2002, 2009, 2012, 2014, and 2019. Notably, the frequency of such co-occurrences increases substantially after 1998, particularly for SAM/La Niña events (seven events post-1998 vs. one before). This may be partly related to the recent increase in multi-year La Niña events, as Wang *et al.* [45] showed that five of six La Niña events after 1998 were multi-year events and that La Niña years have become significantly more frequent than El Niño years. Thus, persistent tropical Pacific forcing may have favored more frequent positive SAM/La Niña co-occurrences through PSA teleconnections. In addition, several post-1998 negative SAM/El Niño events were associated with central-Pacific (CP) rather than eastern-Pacific (EP) El Niño, which is consistent with a late-1990s EP-type shift toward CP-type forcing that favors PSA-like teleconnections [33]. The composite circulation patterns (Fig. S5) further confirm that positive SAM/La Niña events are accompanied by an active PSA pattern and a strengthened ASL, whereas the opposite holds for negative SAM/El Niño events. In contrast, in-phase combinations (i.e. positive SAM/El Niño or negative SAM/La Niña events) yield weaker responses without a clear wave train. The record-low summer Antarctic SIEs in 2022 and 2023 were preceded by strong positive SAM/La Niña events in the preceding austral spring. This enhanced synchronization may help explain the stronger SAM–PSA–ASL coupling across the South Atlantic–South Pacific sector, given the well-established role of ENSO as a key driver of PSA variability [10,41].

**Figure 6.**
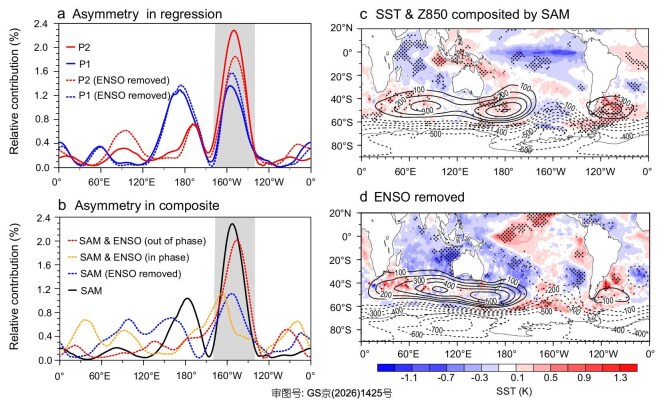
(a) Longitudinal distribution of the relative contribution (%) of the asymmetric component in spring SLP anomalies regressed onto the SAM index for the two periods. Solid lines denote the original series; dashed lines indicate the results after removing the linear ENSO signal via partial regression. The shaded area marks the Amundsen Sea sector (220°E–280°E). (b) Asymmetric contribution of spring SLP anomalies composited under different SAM–ENSO phase combinations. Red line: out‑of‑phase composites (positive SAM/La Niña and negative SAM/El Niño events); orange line: in‑phase composites; black line: composite of all SAM anomaly events; blue line: SAM-only composite after excluding ENSO-related events. (c) SON 850‑hPa geopotential height (contours; gpm) and SST (shading; K) composited for positive and negative SAM events. (d) as in (c), but for anomalies after removing ENSO-related events. The anomalies here are all computed relative to the 1979−2023 climatology.

To further quantify the longitudinal structure of the SAM-related asymmetry, we diagnose the zonally asymmetric component of the SAM-related spring SLP anomalies as a function of longitude for periods P1 and P2 (Fig. 6a). The largest contributions are concentrated over the Amundsen Sea sector (220°E–280°E), indicating that the ASL dominates the asymmetry. In this region, the relative contribution of the asymmetric component increases by 24.2% from P1 to P2. After removing the linearly represented ENSO signal via partial regression for each period, the asymmetric contribution over the Amundsen Sea decreases by 10.8% in P2. This result suggests that, from a linear perspective, ENSO explains only a limited portion of the enhanced zonal asymmetry in the spring SAM pattern.

However, the influence of ENSO on the SAM is not purely linear but also depends critically on their phase relationship, as discussed above. We therefore separate events according to the combined SAM−ENSO phase (Fig. 6b). The out-of-phase events yield the largest asymmetric signal over the Amundsen Sea, comparable in magnitude to the composite of all SAM anomaly events. Removing ENSO-related years from this specific composite reduces the asymmetric component by approximately 22%. The corresponding circulation fields (Fig. 6c and d) show a clear decrease in both the PSA wave train and the ASL intensity after ENSO removal. Together, the results from both the linear removal and the event-screening methods consistently demonstrate that enhanced out‑of‑phase ENSO–SAM combinations, rather than SAM variability alone, play a key role in strengthening the asymmetric SAM‑related circulation over the Amundsen Sea.

## CONCLUSION AND DISCUSSION

This study reveals a nonstationary lagged relationship between the spring SAM and the subsequent summer Antarctic sea ice. Using Rn-EOF and k-means clustering, we identify an interdecadal shift in the spatial pattern of the spring SAM around 1998. Before this shift, the spring SAM is only weakly correlated with the summer Antarctic SIE. After 1998, the SAM develops a stronger PSA-like teleconnection and a deeper ASL, thereby establishing a much stronger linkage with summer sea ice, particularly in the Weddell and Ross Seas. This linkage operates through two distinct regional pathways. In the Weddell Sea, active PSA-related circulation anomalies alter surface heat fluxes, generating persistent warm SST anomalies that favor summer sea-ice melt. In the Ross Sea, despite offshore SST cooling, the ASL-driven wind anomalies enhance offshore sea-ice export in spring, exposing near-coastal open water and activating the ice–albedo feedback that accelerates upper-ocean warming and subsequent summer sea-ice loss. This regional contrast highlights the importance of non-annular SAM components: Weddell Sea changes are dominated by air–sea heat-flux-driven thermodynamic warming, whereas Ross Sea changes are dominated by wind-driven dynamic ice transport, further amplified by the ice–albedo feedback (Fig. 5).

This observed interdecadal transition in the spring SAM pattern is likely modulated by ENSO. The increased frequency of co-occurring positive SAM and La Niña events after 1998 provides more frequent forcing from the tropical Pacific, projecting a stronger PSA-like pattern onto the SAM and thereby enhancing its influence on Antarctic sea ice. Notably, the record-low sea ice observed in February 2022 and 2023 coincided with historically high preconditioning SAM values alongside persistent La Niña conditions. This concurrence highlights combined extreme SAM and La Niña events as key precursors to unprecedented sea-ice losses, further supporting previous findings [14].

These findings provide a mechanistic, observation-based framework for interpreting recent Antarctic sea-ice extremes. Because most current climate models fail to capture Antarctic sea-ice trends and variability [46], we adopt an observational perspective for mechanistic understanding. The identified nonstationary SAM–PSA–sea ice linkage, regulated by ENSO, offers a physical basis for improving model representation and future projections. Specifically, two aspects require improvement: (1) more accurate simulation of the wave-like (or zonally asymmetric) features in the SAM components, especially over the Weddell and Ross Seas, where they are central to the two-pathway mechanism; and (2) improved representation of phase-synchronized SAM–ENSO variability, especially the increased frequency of positive SAM/La Niña events after 1998, which strengthens the PSA-like wave train and the lagged SAM influence on summer sea ice.

However, the causes of the enhanced synchronization between the SAM and La Niña since the late 1990s remain uncertain and warrant further investigation. It may reflect modulation by low-frequency Pacific variability, particularly the Interdecadal Pacific Oscillation phase shift in the late 1990s, which alters ENSO‐related climate anomalies across the Southern Hemisphere [33,47–49]. Alternatively, anthropogenic forcing has accelerated Southern Ocean warming and may have shifted the Antarctic climate system into a new regime [7], which could fundamentally alter tropical–extratropical interactions across the Southern Hemisphere. Definitive attribution will require longer records and model simulations, as our observational analysis cannot formally distinguish internal variability from externally forced change. Both proposed mechanisms therefore warrant further investigation.

## MATERIALS AND METHODS

### Data

Daily and monthly Antarctic SIC and daily sea ice velocity were obtained from the National Snow and Ice Data Center, with a resolution of 25 km × 25 km [50,51]. The SIE was derived by aggregating the areas of all pixels whose SIC equaled to or exceeded 15%. Monthly mean atmospheric and oceanic data, including geopotential height, wind fields, SLP, surface heat fluxes, and SST, were acquired from the European Centre for Medium-Range Weather Forecasts (ECMWF) ERA5 reanalysis products with a 1.5° × 1.5° horizontal resolution [52]. The analysis period spans from 1979 to 2023.

### Statistical analysis

We employ EOF analysis of SLP anomalies south of 20°S to characterize the spring SAM pattern and associated index, following the methodology of Thompson *et al.* [53]. To capture its temporal evolution, we implement the running-EOF (Rn-EOF) method introduced by Zhang *et al.* [54], which utilizes sliding time windows to detect nonuniform SLP changes. For comparison, we also use the station-based SAM index defined by Marshall [20]. ENSO variability is quantified by the Niño 4 index (5°S–5°N, 160°E–150°W) [55], which is more strongly linked to Antarctic sea ice than to Niño 3.4 or Niño 3 [56]. To objectively classify SAM spatial patterns, we apply k-means clustering, a method widely used to categorize atmospheric circulation regimes [57]. The optimal number of clusters is determined using the silhouette score (S), which reflects cluster compactness, and the SSEs [58], which reflects inter-cluster separation. This combined methodology enables robust identification of distinct SAM pattern classes on the basis of objective similarity criteria.

The zonally asymmetric component is obtained by removing the zonal-mean anomaly at each latitude from the spring SLP pattern regressed onto the SAM index or composited by SAM and ENSO phase. For each longitude, we then compute the latitude-area–weighted mean square of this asymmetric component over the Southern Hemisphere (90°–20°S). These values are subsequently normalized by their sum across all longitudes and expressed as percentages, yielding the relative contribution of each longitudinal sector to the total zonally asymmetric variance. This diagnostic highlights the longitudinal sectors that dominate the non-annular component of the SAM-related circulation.

### Sea-ice budget analysis

To quantify the relative contributions of dynamic and thermodynamic processes to SAM-related sea-ice anomalies, we apply a SIC budget analysis following Holland and Kimura [59]:


(1) \begin{eqnarray*}
\frac{{\partial C}}{{\partial t}} &=& - \nabla \cdot \left( {{\boldsymbol{u}}C} \right) + {{f}_c} - r\\
&=& {\mathrm{\ }} - {\boldsymbol{u}} \cdot \nabla C - C \cdot \nabla {\boldsymbol{u}} + {{f}_c} - r,
\end{eqnarray*}

where ∂*C*/∂*t* is the ice tendency, *C* is the sea ice concentration, and ***u*** is the sea ice drift. The term $- \nabla \cdot ( {{\boldsymbol{u}}C} )$ represents the dynamic process, including advection ($- {\boldsymbol{u}} \cdot \nabla C$) and divergence ($- C \cdot \nabla {\boldsymbol{u}}$). The term *f_c_* represents the thermodynamic process of melting/freezing. Mechanical redistribution processes (*r*), including ridging and rafting, are assumed to be small relative to thermodynamic changes and are therefore neglected [40].

The SIC tendency is calculated daily using a central difference in time. The advection and divergence terms are also computed daily using central differences in space and then averaged over the same 3-day interval to maintain a consistent time stamp with the tendency term. To reduce noise, sea-ice drift fields are smoothed using a 7 × 7 grid-cell square-window filter before calculating the dynamic term. The thermodynamic contribution is obtained as the residual of the budget equation. Daily budget terms are then averaged to monthly and seasonal means. Because sea-ice drift is less reliable near the ice edge, the budget decomposition is most robust within the interior ice pack [40].

## Supplementary Material

nwag314_Supplemental_File

## Data Availability

All data supporting this study are publicly available: sea ice concentration and ice drift data are obtained from NSIDC (https://nsidc.org/data/g02202/versions/4 and https://nsidc.org/data/nsidc-0116/versions/4); ERA5 reanalysis data are sourced from Copernicus Climate Data Store (https://cds.climate.copernicus.eu/datasets); and the Marshall SAM index is obtained from the British Antarctic Survey (http://www.nerc-bas.ac.uk/icd/gjma/sam.html). All analyses were performed via publicly available software including NCAR Command Language (NCL v6.6.2) and Python.
