# Supplementary material for: Interdecadal shift in the spring Southern Annular Mode intensifies its lagged influence on Antarctic summer sea ice
Source: Natl Sci Rev. 2026 May 27;13(11):nwag314. doi: 10.1093/nsr/nwag314 (PMC13289740; doi:10.1093/nsr/nwag314)
Supplement: nwag314_Supplemental_File [file nwag314_supplemental_file.docx]

Supplementary Materials for

**Interdecadal Shift in the Spring Southern Annular Mode Intensifies Its Lagged Influence on Antarctic Summer Sea Ice**

Juan Dou^1, 2^, Xiangzhou Song^1, 2*^, and Renhe Zhang^3^

^1^Key Laboratory of Marine Hazards Forecasting, Ministry of Natural Resources, Hohai University, Nanjing 210098, China.

^2^College of Oceanography, Hohai University, Nanjing 210098, China.

^3^Department of Atmospheric and Oceanic Sciences and Institute of Atmospheric Sciences, Fudan University, Shanghai 200438, China.

*Corresponding author: Xiangzhou Song ([xzsong@hhu.edu.cn)](mailto:email@address.edu))

**Contents of this file**

Figures S1–S5

******

**Figure S1.** Running-EOF analysis of SLP south of 20°S based on a 21-year sliding window. The year 1989 indicated in the figure represents the 21-year sliding window (1979–1999) centered around 1989, and so forth. The blue number in the upper right corner of each figure indicates the category number.

**Figure S2.** Monthly lagged correlation coefficients of the total Antarctic sea-ice extent (SIE) with the preceding SON SAM index during P2 (1998–2023). Months are shown from December (lag 1 month) to the following November (lag 11 month). The dashed lines indicate the 95% significance level.

**Figure S3.** Same as Figure 4, but for the correlations with the standardized SON SAM index during P1 (1979–1997).

**Figure S4.** Regression of SONDJF (September to February) sea-ice budget anomalies onto the spring (SON) SAM index during P2. Panels show (a) total sea-ice concentration tendency (units: % day^−1^), (b) the dynamic component (units: % day^−1^), and (c) the thermodynamic component (units: % day^−1^). Stippling denotes values significant at the 95% confidence level.

**Figure S5.** SST anomalies (shading; K) and 850‑hPa geopotential height (contours; gpm) during SON under different SAM–ENSO phase combinations. Left panels show out‑of‑phase composites: (a) negative SAM/El Niño, (b) positive SAM/La Niña, and (c) their difference (a minus b). Right panels show in‑phase composites: (d) positive SAM/El Niño, (e) negative SAM/La Niña, and (f) their difference (d minus e). Black dots denote SST anomalies that are significant at the 95% confidence level. The anomalies here are all computed relative to the 1979−2023 climatology.
